# Changes in Carcass Condemnation During a Six-Year Transition from Antibiotic-Based to Antibiotic-Free Broiler Production in Thailand: A Bayesian Structural Time-Series Analysis

**DOI:** 10.3390/ani16132050

**Published:** 2026-07-03

**Authors:** Veerasak Punyapornwithaya, Supitchaya Siriyakhun, Chalita Jainonthee, Duangporn Pichpol, Pranee Pirompud, Panneepa Sivapirunthep, Chanporn Chaosap

**Affiliations:** 1 Research Center for Veterinary Biosciences and Veterinary Public Health, Faculty of Veterinary Medicine, Chiang Mai University, Chiang Mai 50100, Thailand; veerasak.p@cmu.ac.th (V.P.); siriyakhun.s@gmail.com (S.S.); chalita.j@cmu.ac.th (C.J.); duangporn.p@cmu.ac.th (D.P.); 2 Veterinary Public Health and Food Safety Center for Asia Pacific, Faculty of Veterinary Medicine, Chiang Mai University, Chiang Mai 50100, Thailand; 3 Sun Group Company, Jathujak, Bangkok 10900, Thailand; pirompud@gmail.com; 4 Department of Agricultural Education, School of Industrial Education and Technology, King Mongkut’s Institute of Technology Ladkrabang, Bangkok 10520, Thailand; panneepa.si@kmitl.ac.th

**Keywords:** antibiotic-free rearing, carcass condemnation, Bayesian structural time-series, counterfactual analysis, commercial broilers

## Abstract

The transition from antibiotic-based (AB) to antibiotic-free (ABF) broiler production represents a major shift in poultry management, with potential implications for flock health, welfare, and processing outcomes. This study evaluated its impact on condemnation percentage (%condemnation) using Bayesian structural time-series (BSTS) analysis. Data from a Thai integrator comprised 105,899 truckload-level records (2015–2020) across 260 contract farms. The AB period (2015–2017) served as the baseline, and the ABF period (2018–2020) was assessed using counterfactual projections. Time-series decomposition and change-point analysis revealed an increasing trend in %condemnation during the early phase of ABF implementation, followed by a decline in 2020, with five structural shifts detected. The BSTS model estimated an absolute effect of +1.10% (95% CI: −1.50 to 3.80; *p* = 0.207) and a relative effect of +95% (95% CI: −38% to 657%), indicating no statistically significant causal impact. The transient increase may reflect short-term adaptation challenges, whereas subsequent stabilization may be associated with adaptation to ABF production and other concurrent management changes. Overall, the transition from AB to ABF production did not significantly affect %condemnation. Adaptive management measures were implemented as a company-wide policy but were not directly evaluated within the BSTS framework.

## Simple Summary

The shift from antibiotic-based (AB) to antibiotic-free (ABF) broiler production is an important change in poultry farming that may affect animal health and processing outcomes. This study analyzed data from 260 farms in Thailand to determine whether this transition influenced the rate of carcass condemnation. The results showed that condemnation rates increased slightly during the early stage of ABF implementation but later declined, with no overall significant impact of the transition. These temporary changes likely reflect short-term adjustment challenges, while later stabilization suggests improvements in farm management, biosecurity, and alternative health strategies. Although company-wide adaptive management measures were introduced during this transition, their specific effects were not directly evaluated. Overall, the findings indicate that moving to antibiotic-free production can be achieved without negatively affecting condemnation rates.

## 1. Introduction

The global poultry sector has increasingly shifted away from routine prophylactic antibiotic use toward antibiotic-free (ABF) production systems in response to consumer concerns regarding antimicrobial resistance, regulatory changes, and demand for ABF-labelled products [1,2,3,4]. Although reducing antibiotic use aligns with public health and animal welfare objectives, the withdrawal of antibiotics may create challenges for flock health and production performance under intensive farming conditions [3,5,6].

Although ABF production is promoted to reduce antimicrobial resistance and meet consumer demand, previous studies have reported mixed effects on poultry health and production performance. Antibiotic withdrawal may increase susceptibility to enteric diseases, mortality, and production variability, particularly when management and biosecurity are inadequate, and some studies have also reported higher incidences of dermatitis, systemic infections, and carcass defects in ABF systems, which may contribute to increased carcass condemnation [7,8]. However, effective management, nutrition, vaccination, and biosecurity strategies can help mitigate these challenges and support successful ABF production. These contrasting findings highlight the need for further evaluation of condemnation outcomes during the transition from AB to ABF production under commercial conditions.

Carcass condemnation represents a substantial cause of economic loss and food waste because carcasses or parts are removed at post-mortem inspection [9]. Typical reasons include contamination, traumatic injury and pathology such as septicemia, dermatitis, pericarditis and ascitic syndrome [9,10,11,12]. Variation in the percentage of carcass condemnation (%condemnation) is also influenced by bird- and system-level factors, including breed, health, body weight, age, weather, stocking density, in-transit factors and pre-slaughter handling [13,14,15]. A previous data-driven study in Thailand indicates that mean body weight, weight per crate, mortality and culling rates, and lairage time are strong predictors of higher %condemnation [16]. Given these complex interactions, shifts in production systems from AB to ABF can further affect condemnation outcomes. Understanding these changes requires an integrated perspective that considers not only farm-to-slaughterhouse management factors but also post-mortem processing conditions that determine the final inspection outcome.

Bayesian structural time-series (BSTS) analysis provides a principled framework for evaluating interventions in longitudinal production data by generating counterfactual trajectories for outcomes if the intervention had not occurred [17,18,19]. It has been applied across agricultural economics, public health and animal production to appraise policy and management changes [20,21,22,23,24]. In the present study, BSTS was applied to a vertically integrated broiler production system in which the company operates a large network of contract farms managed under standardized protocols. The transition from AB to ABF production was implemented simultaneously across all farms as a coordinated policy shift, providing a suitable setting for BSTS analysis. This uniform intervention across a broad population of farms allows BSTS to isolate policy-related effects from background variation and long-term trends, offering a data-driven evaluation of the ABF transition under commercial conditions in Thailand.

In Thailand, antibiotics as growth promoters are prohibited, and broilers produced for export to European Union (EU) markets must be raised without antibiotic use throughout the production cycle. In contrast, therapeutic antibiotic use remains permitted in some domestic production systems under veterinary supervision. Because the complete withdrawal of antibiotics may influence flock health and processing outcomes, evaluating condemnation trends during the ABF transition is important for understanding the broader production implications of this management change.

Previous BSTS applications in poultry production primarily focused on dead-on-arrival (DOA) data associated with transport-related mortality [24]. Expanding this approach to %condemnation provides a broader assessment encompassing both transport- and processing-related outcomes. 

Furthermore, studies examining the effect of transitioning from AB to ABF systems on carcass condemnation remain very limited, despite growing interest in this topic among producers, veterinarians, and policymakers. Addressing this gap provides relevant evidence for a broad audience concerned with sustainable and responsible poultry production.

The objective of this study was to determine the effect of transitioning from AB to ABF broiler production on %condemnation in commercial farms in Thailand using BSTS analysis. By analyzing longitudinal data before and after the transition, this study provides evidence regarding condemnation trends following the implementation of ABF production under commercial conditions in Thailand.

## 2. Materials and Methods

**Figure 1:**
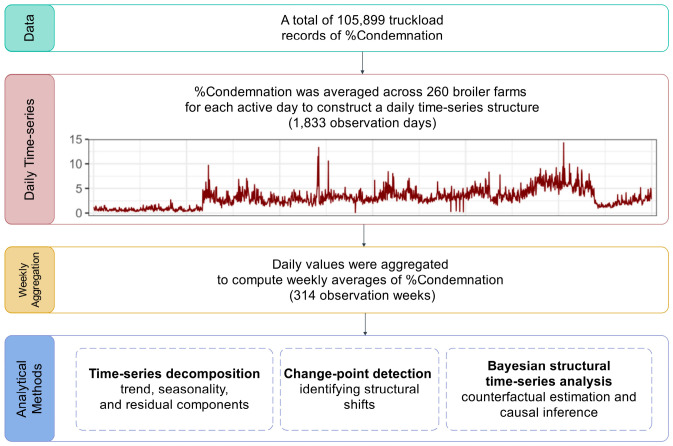
Data and analytical workflow. The dataset comprised 105,899 truckloads from 260 broiler farms with recorded %condemnation at a single slaughterhouse. Truckload values were averaged by day to form a daily time series and subsequently aggregated into weekly observations including 314 observation weeks. Analyses included time-series decomposition, change-point detection, and Bayesian structural time-series analysis.

The study followed a structured workflow comprising data definition and management, descriptive analysis, time-series visualization, change-point detection, and BSTS analysis. The overall analytical framework is illustrated in Figure 1.

### 2.1. General Overview of Broiler REARING Practices

The production system has been described previously in detail [24]. The flocks primarily consisted of commercial fast-growing Ross 308 broilers. Target slaughter age ranged from 40 to 49 days, with market weights of 2.6–3.5 kg. Birds were housed in climate-controlled, tunnel-ventilated barns equipped with evaporative cooling pads and automated fans. Initial brooding temperatures (30–32 °C) were gradually reduced to 26–28 °C, depending on flock age and environmental conditions. Stocking densities were maintained at 10–12 birds/m^2^ (28–41 kg/m^2^). Lighting schedules included 23L:1D for the first 7 days, followed by 18L:6D. Diets were based on corn–soybean formulations designed to meet Ross 308 nutritional requirements [25]. Vaccination programmes included Newcastle Disease (ND) and Infectious Bronchitis (IB) spray vaccines, an immune-complex vaccine for Infectious Bursal Disease (IBD), and a recombinant HVT-ND vaccine administered subcutaneously on day 1. Booster ND + IB vaccines were provided via drinking water at 7–9 days of age. Coccidiosis control was achieved through a rotation programme using ionophores and chemical coccidiostats.

Under the AB programme, two antibiotic classes were commonly administered. Macrolides (e.g., tilmicosin, 10–15 mg/kg BW for 3–5 days) were used during the early production period, whereas tetracyclines (e.g., doxycycline, 10–20 mg/kg BW for 3–5 days) were administered when clinical signs appeared. Mandatory withdrawal periods (7–14 days) were observed before slaughter. In the ABF system, no antibiotics were used at any stage. Instead, alternative strategies were implemented, including phytogenic feed additives (e.g., oregano oil, carvacrol, thymol at 100–150 ppm), organic acids (e.g., propionic, benzoic, and acetic acids in drinking water at 0.1–0.2%), and probiotics (*Lactobacillus* and *Bacillus* spp.). These non-antibiotic interventions are widely recognized as alternatives to support gut health and pathogen control [6]. The intervention evaluated in this study was the company-wide transition from conventional antibiotic-based production to an ABF production system in 2018. This transition involved not only the removal of antibiotics but also the concurrent implementation of alternative feed additives including phytogenic feed additives, organic acids delivered via drinking water, and probiotic preparations; enhanced biosecurity measures; environmental management practices; and welfare-oriented management strategies.

### 2.2. Handling, Transport, Slaughter Procedures, and Condemnation Recorded

The entire flock was collected in a single catch for slaughter, with no thinning practice. The downtime between flocks before introducing a new batch was 21–24 days, allowing for approximately 5.5 production cycles per year. The average production performance of the birds was as follows: they had a daily weight gain of 68–70 g/day, feed conversion ratio of 1.55–1.60, and a mortality rate of 3–5% during the production cycle. Birds were sent to the processing plant at 40–49 days of age with a final body weight of approximately 2.8–2.9 kg. Bird handling and transport followed protocols described by [24]. Feed and water were withdrawn ~3 h before loading. Birds were manually caught and placed in crates at 4–10 birds per crate (0.72 × 0.54 × 0.31 m; maximum ~18 kg load). A full load on each truck consisted of 480–495 crates arranged in stacks before loading onto trucks. Transport distances ranged from 25 to 354 km. To minimize heat stress, feathers were dampened with water spray before loading, and during transit, trucks were sprayed every 150 km. Transport occurred under typical Thai conditions (28–35 °C; 70–90% humidity). At the slaughterhouse, trucks were held in ventilated lairage areas for 30–120 min with evaporative cooling. Mortality was recorded at unloading. Birds were stunned (800 Hz, 50–100 V, 10 s) before slaughter. After slaughtering, birds were automatically eviscerated, with carcasses and internal organs conveyed together along the line. Inspectors examined the line and removed any condemned carcasses or organs. Reasons for condemnation included purulent abscesses, cellulitis, arthritis, viscera deemed unfit for consumption, cachexia, emaciation, abnormal carcass coloration, and carcasses lacking offal.

### 2.3. Truckload and Condemnation Data

This analysis uses the same source population and study period previously reported for the dead-on-arrival (DOA) evaluation but focuses on post-mortem rejection at processing rather than transport mortality [24]. Data were provided by a large Thai integrator operating 250–280 contract farms that implemented a company-wide transition from AB to ABF rearing. The observational unit was the truckload delivered to a single processing plant. Condemnation was expressed as a percentage, calculated by dividing the number of broilers condemned per truck at the processing plant by the total number of broilers transported from that truck, and then multiplying the result by 100.

For each truckload, the %condemnation was defined as

$$C_{\mathrm{id}}=\frac{c_{\mathrm{id}}^{\mathrm{condemned}}}{N_{\mathrm{id}}}\times 100,$$

where $c_{\mathrm{id}}^{\mathrm{condemned}}$ is the number of condemned birds and $N_{\mathrm{id}}$ is the total number of birds from truckload i slaughtered on day d at the single processing plant [9,15]. An overview of data flow and analysis steps is shown in Figure 1.

### 2.4. Time-Series Data of %Condemnation

The dataset comprised 105,899 daily truckload records collected from 260 contract farms over six calendar years, covering three years before (2015–2017) and three years after (2018–2020) the transition from AB to ABF. The %condemnation for each truckload was calculated as defined above. To construct the analytical series, these truckload-level values were aggregated at the weekly level. For each week w, the average %condemnation was computed as

$${\bar{C}}_{w}=\frac{1}{n_{w}}\sum_{i=1}^{n_{w}}C_{\mathrm{iw}},$$

where ${\bar{C}}_{w}$ is the average %condemnation in week w, $C_{\mathrm{iw}}$ is the truckload-level percentage for truckload i in week w, and $n_{w}$ is the number of truckloads processed that week. This yielded 314 weekly observations structured as a univariate time series indexed by week (t = 1, 2, …, 314). All records originated from verified processing-plant systems and were treated as genuine commercial variability. For visualization only, truckload counts and %condemnations were summarized by month to illustrate within-year and seasonal patterns.

### 2.5. Statistical Analysis

#### 2.5.1. Time-Series Decomposition and Change-Point Analysis

The weekly %condemnation time series was decomposed into trend, seasonal, and irregular components using classical seasonal decomposition. This approach was used to distinguish long-term changes associated with production system transitions from recurring seasonal patterns and random variation. The BSTS analysis was implemented as a univariate model using weekly slaughterhouse condemnation rates as the response variable. Potential covariates, including farm-level management, flock health, stocking density, breed, feed formulation, and processing-related factors, were not included because consistent longitudinal data for these variables were unavailable across the study period. Seasonal variation was assessed using Friedman’s rank-sum test, a non-parametric procedure for detecting differences across repeated time periods.

To evaluate structural changes in condemnation dynamics, change-point detection was performed using the *cpt.meanvar* function in the *changepoint* package [26] in R version 4.5.1 [27]. This method jointly tests shifts in both mean and variance of the time series. The binary segmentation algorithm was applied to recursively partition the series into sub-segments and identify multiple change-points by maximizing a penalized likelihood criterion [26].

#### 2.5.2. Bayesian Structural Time-Series (BSTS) Analysis

The effect of transitioning to ABF production on %condemnation was evaluated using a BSTS framework. The BSTS generates counterfactual predictions representing the expected outcome in the absence of the intervention and quantifies the difference between observed and predicted values over time [28]. The BSTS framework accounts for latent trends, stochastic variation, and unobserved temporal dynamics while providing posterior inference on pointwise and cumulative effects, making it suitable for longitudinal intervention studies. The general BSTS model follows the standard-space formulation described by Brodersen et al. (2015) [17], which can be expressed as

$$y_{t}=Z_{T}^{t}\alpha_{t}+\varepsilon_{t}$$

$$\alpha_{t+1}=T_{t}\alpha_{t}+R_{t}\eta_{t}$$

$$\varepsilon_{t}\sim N(0,\sigma_{t}^{2}),\;\eta_{t}\;∼\;N(0,Q_{t}),$$

where $y_{t}$ denotes the %condemnation at time ${t,\;\alpha }_{t\;}$ is the latent state vector capturing local level, trend, and seasonal components, and $\varepsilon_{t}\sim N(0,\sigma_{t}^{2})$ represents the observation error. The term $T_{t}$ is the transition matrix that governs the temporal dynamics of the state, $R_{t}$ is the control matrix linking state disturbances, and $\eta_{t}\;∼\;N(0,Q_{t})$ is a vector of state errors with variance–covariance matrix $Q_{t}$. The design matrix $Z_{t}$ links the latent state to the observed outcome, while the state vector $\alpha_{t}$ captures unobserved components such as the local level, slope (trend), and seasonal structure.

The model was estimated within a Bayesian framework by specifying prior distributions on regression coefficients, state variances, and observation noise. Posterior inference was conducted via Markov chain Monte Carlo (MCMC) sampling, which provides predictive distributions rather than point estimates, thereby allowing for direct quantification of uncertainty around causal effects [17].

Counterfactual outcomes, defined as the expected %condemnation if antibiotics had not been withdrawn, were derived from the posterior predictive distribution. The pointwise causal effect at time t [17] is computed as

$$\phi_{t}=y_{t}^{\mathrm{obs}}-{\tilde{y}}_{t},$$

where $y_{t}^{\mathrm{obs}}$ is the observed %condemnation and ${\tilde{y}}_{t}$ is the counterfactual predicted by the model.

The cumulative effect over the post-intervention period T, representing the total change in %condemnation across all time points following the intervention, is given by

$$\sum_{t\in T}\phi_{t}=\sum_{t\in T}\left(y_{t}^{\mathrm{obs}}-{\tilde{y}}_{t}\right)$$

The term $\sum_{t\in T}\phi_{t}$ represents the cumulative causal effect of transitioning from AB to ABF production on %condemnation across the post-intervention period. It is obtained by summing the pointwise effects ($\phi_{t}$) of each time point t within the post-intervention period T. Each effect is defined as the difference between the observed outcome $y_{t}^{\mathrm{obs}}$ and the counterfactual prediction ${\tilde{y}}_{t}$, representing what would have occurred had antibiotics remained in use. Thus, it can be said that $\sum_{t\in T}\phi_{t}$ quantifies the total impact of ABF adoption on processing outcomes over time. Additionally, to provide a normalized summary measure, the average effect is expressed as [17]

$$\bar{\phi }=\frac{1}{\left|T\right|}\sum_{t\in T}\left(y_{t}^{\mathrm{obs}}-{\tilde{y}}_{t}\right)$$

The term $\bar{\phi }$ denotes the average causal effect of the intervention across the post-intervention period, while |t| represents the number of time points within that period.

A positive average effect ($\bar{\phi }$) indicates that observed condemnation rates were higher than the counterfactual prediction, whereas a negative value indicates lower condemnation rates than expected under the counterfactual scenario. Values closer to zero indicate minimal deviation from the counterfactual prediction.

Model specification: The BSTS model was implemented as a univariate state-space model using weekly %condemnation as the response variable. The pre-intervention period (2015–2017) was used to estimate the latent temporal structure of the series, and the post-intervention period (2018–2020) was used to evaluate deviations from the counterfactual trajectory following implementation of the antibiotic-free (ABF) programme. The model incorporated latent trend components to capture temporal dynamics in %condemnation and generated posterior predictive distributions of the expected outcome under the hypothetical scenario in which the AB programme had been maintained. No external covariates were included because consistent longitudinal measurements were unavailable across the study period.

Prior assumptions: Model estimation was performed using the Bayesian structural time-series framework implemented in the CausalImpact package (version 1.4.1). Prior distributions for model parameters, including state variances and observation noise, were specified according to the default prior structure of the package. These priors are weakly informative and are intended to regularize parameter estimation while allowing the observed data to primarily determine posterior inference. Given the absence of external regressors, the analysis focused on inference derived from the temporal structure of the observed series.

Convergence diagnostics and sensitivity analysis: Posterior inference was obtained using Markov chain Monte Carlo (MCMC) sampling within the BSTS framework. Model convergence was assessed through visual inspection of posterior sampling outputs and evaluation of the stability of posterior predictive estimates. Formal sensitivity analyses under alternative prior distributions or alternative state-space specifications were not performed.

Data management and visualization were performed using the “*dplyr*” (version 1.2.1) [29] and “*ggplot2*” (version 4.0.3) [18] packages, respectively, in R (version 4.5.1) [30]. Functions from the “feasts” package (version 0.5.0) were used to decompose the % condemnation time-series data, whereas functions from the “*stats*” package (version 4.6.0) were used to perform Friedman’s test. The “*CausalImpact*” package (version 1.4.1) [17] in R was used to implement the BSTS analysis and to generate both quantitative outputs and graphical representations.

## 3. Results

### 3.1. Patterns, Trends, and Seasonality of %Condemnation

**Figure 2:**
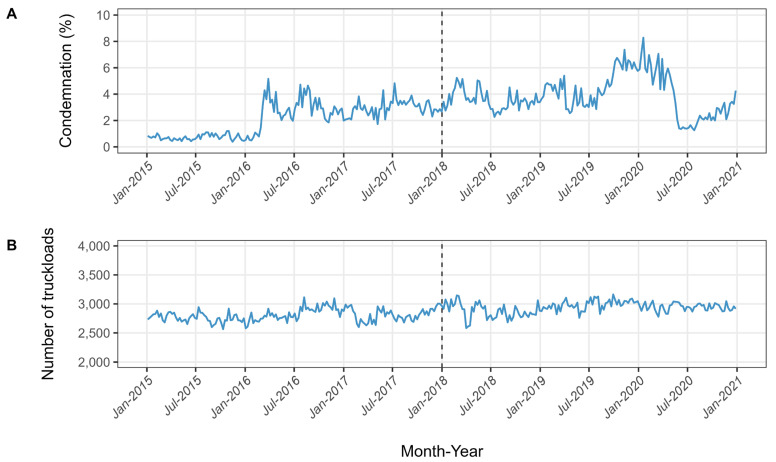
Monthly patterns in truckload volume and percentage of carcass condemnation (%condemnation) before and after the transition to antibiotic-free (ABF) production. (**A**) Number of truckloads processed per month from 2015 to 2020. (**B**) Average monthly %condemnation over the same period. The dashed vertical line marks the 2018 transition, separating the antibiotic-based (AB) period (2015–2017) from the ABF period (2018–2020). Darker shading indicates higher values.

**Figure 3:**
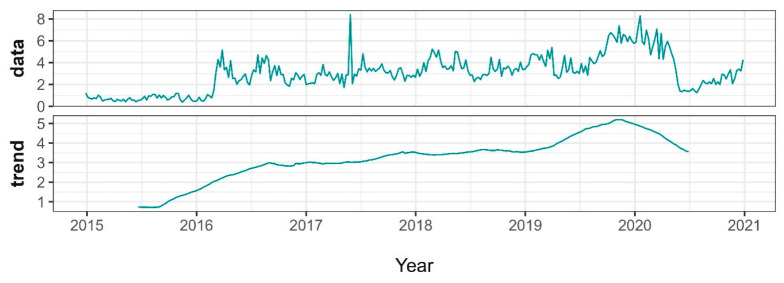
Decomposition of the weekly percentage of carcass condemnation (%condemnation) into its trend component.

Over the study period, 1883 days of transported truckloads were recorded, averaging 56 truckloads per active day. When summarized at the weekly level (314 weeks), the flow averaged 336 truckloads per week. These values reflect observed activity only, as some calendar days and weeks had no truck movements. Averages of weekly truckload and %condemnation each month for AB and ABF periods are shown in Figure 2. Time-series decomposition revealed a progressive increase in %condemnation from 2015 to 2019, followed by a decline in 2020 (Figure 3). Friedman’s rank-sum test indicated no statistically significant seasonal pattern in the data.

### 3.2. Change-Point Analysis of %Condemnation Trends

**Figure 4:**
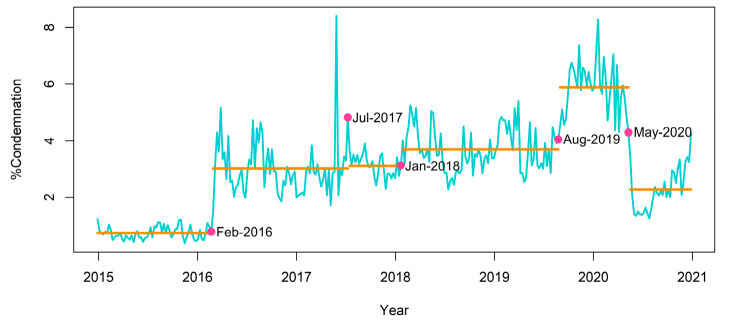
Change-point analysis of the weekly %Condemnation time series (2015–2020). Red points indicate detected change-points; yellow segments show fitted means within segments. The antibiotic-based period (2015–2017) precedes the antibiotic-free period (2018–2020).

Change-point detection identified five shifts in the weekly %condemnation series between 2015 and 2020 (Figure 4), partitioning the data into six segments. The earliest segment (early 2015) showed the lowest levels under the AB programme, followed by a steady rise through 2017. After the ABF implementation in 2018, %condemnation increased further, with the highest levels observed from late 2019 into early 2020, before declining thereafter. These change-points describe structural shifts in the series; causal attribution is examined using the BSTS framework in the subsequent section.

### 3.3. Bayesian Structural Time-Series Analysis of Post-Transition Trends

BSTS provides an estimate of changes temporally associated with the intervention; however, interpretation remains subject to potential confounding from unmeasured factors. The BSTS model compared observed %condemnation during the ABF period with counterfactual values projected from pre-transition trends. The observed average under ABF production was 3.90%, whereas the predicted counterfactual was 2.80% (Table 1), yielding an absolute effect of +1.10 percentage points (95% credible interval −1.50 to 3.80) and a relative effect of +95% (95% credible interval −38% to 657%). The Bayesian one-sided tail-area probability (*p* = 0.207) indicated that this difference was not statistically significant (Table 1). The posterior mean relative effect was estimated at +95%; however, the associated credible interval was wide and included zero, indicating substantial uncertainty and no statistically significant evidence of an intervention effect.

**Figure 5:**
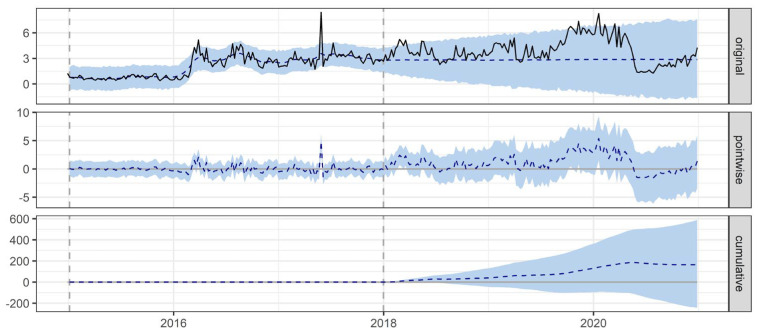
Bayesian structural time-series causal-impact plot for %Condemnation (2015–2020). **Top**: observed series (black) and posterior counterfactual (blue dashed) with 95% credible interval (shaded). **Middle**: pointwise effects (observed − counterfactual) with 95% credible interval. **Bottom**: cumulative effect across the post-intervention period with 95% credible interval. The vertical dashed line marks the 2018 transition to antibiotic-free rearing.

Graphical outputs (Figure 5) display the observed and predicted weekly values (top panel), the pointwise differences (middle panel), and the cumulative differences (bottom panel); the vertical dashed line marks the start of the ABF programme. The pointwise differences represent the estimated weekly deviation between the observed and counterfactual condemnation rates following ABF implementation, while the cumulative differences show the aggregated effect over time, allowing for assessment of whether deviations persist or are offset across the post-intervention period.

## 4. Discussion

This study examined changes in %condemnation before and after the implementation of ABF systems, providing insight into management performance and processing outcomes under commercial production conditions.

### 4.1. Trend, Seasonality, and Change-Points

The upward trend observed during the early ABF phase is biologically plausible, as antibiotic withdrawal may initially disrupt microbial balance and challenge disease control, thereby increasing susceptibility to infectious or inflammatory conditions. As production systems adjusted to the new management framework, a gradual decline in %condemnation emerged, reflecting adaptation supported by enhanced biosecurity, improved husbandry, and the use of non-antibiotic health-support measures. This adaptive trajectory corresponds with the identified change-points, which illustrate short-term instability followed by progressive stabilization as ABF practices became embedded in routine management. In addition, small early-year fluctuations were observed in some years; however, time-series decomposition did not identify a consistent or statistically significant seasonal pattern across the study period. These findings may be attributed to seasonal stressors such as temperature and humidity fluctuations, which are known to predispose broilers to respiratory or metabolic disorders [31].

When compared with the %DOA findings reported for the same production system [24], the BSTS analysis indicated no significant long-term impact of ABF adoption on %DOA, despite a transient increase during early transition, highlighting the importance of adaptive management and offering practical guidance for ABF implementation in Thailand. In this study, the temporal pattern of %condemnation showed similar overall trends and seasonal characteristics. Both indicators exhibited early increases coinciding with the initial implementation of ABF practices, followed by gradual declines as management stabilized. The pattern of multiple change-points observed in both datasets further suggests that transitional fluctuations were part of a broader systemic adjustment rather than random variation. Seasonal peaks were also aligned, with increases during cooler months linked to environmental stressors such as temperature and humidity fluctuations that predispose broilers to respiratory or metabolic disorders [32]. Despite a cooler season in Thailand (November–February), no recurring seasonal pattern in condemnation rates was detected across years. Because the analysis focused on total condemnation percentage, potential differences among specific condemnation categories could not be evaluated. Aggregating multiple condemnation causes into a single outcome may mask category-specific responses associated with infectious, metabolic, welfare-related, or processing-related factors. Future studies examining temporal trends in individual condemnation categories may provide greater biological insight into the effects of ABF production systems.

### 4.2. Impact of ABF Transition on %Condemnation

Although a numerical increase in %condemnation was observed during the early ABF phase, the BSTS analysis indicated that these fluctuations were not statistically different from the counterfactual scenario. An important limitation of this study is the absence of covariates describing flock health, environmental conditions, stocking density, feed formulation, management practices, and processing factors. Consequently, the BSTS model was based solely on the temporal pattern of condemnation outcomes and could not account for all potential confounders that may have changed during the study period. Therefore, the estimated effects should be interpreted as associations coinciding with the transition to the ABF production system rather than definitive causal effects attributable to the intervention. This finding suggests that the observed variations were more consistent with underlying long-term trends and random fluctuations than with a direct causal effect of antibiotic withdrawal. This finding aligns with the change-point results, which also showed temporary instability followed by stabilization as production adapted to new management conditions. Although transient increases in condemnation percentage were observed during the early post-transition period, the BSTS analysis did not identify a statistically significant overall effect of the ABF production system transition. Therefore, biological explanations related to immune function, disease susceptibility, or adaptation processes should be interpreted cautiously. The observed decline in condemnation after 2019 may be consistent with management adaptations commonly implemented in ABF systems. Probiotics, phytogenics, enhanced biosecurity, environmental management, and welfare-related practices were implemented as part of the ABF production programme, and the present study was not designed to evaluate the effectiveness of these individual interventions. The explanations should be considered hypotheses rather than confirmed mechanisms underlying the observed patterns. Therefore, their potential contributions to the observed condemnation patterns remain speculative and should be interpreted with caution.

From a biological and management perspective, the temporary increase observed after the ABF transition is plausible, as the withdrawal of antibiotics may initially increase susceptibility to health challenges before adaptation to the new production system occurs. During this transition period, enhanced biosecurity measures were implemented, including stricter sanitation procedures, footbath use, visitor access restrictions, and downtime requirements between flocks. These measures were intended to reduce pathogen introduction and transmission, thereby supporting flock health under ABF production conditions. The removal of prophylactic antimicrobials can temporarily reduce protection against subclinical and opportunistic infections, increasing birds’ susceptibility to inflammatory and systemic conditions such as cellulitis, arthritis, and septicemia [9,33,34,35]. This heightened vulnerability is most evident during the early stages of ABF implementation, before alternative preventive measures are fully optimized. However, the lack of sustained deterioration in condemnation rates indicates that farms were able to adapt effectively. Enhanced management practices, including stricter biosecurity, improved litter and water quality, optimized ventilation [36], and the adoption of non-antibiotic health supports such as probiotics [36], organic acids [37,38], and phytogenics [39,40], likely contributed to stabilization over time. Furthermore, comparable patterns were previously observed in the DOA analysis within the same production system [24], where transient increases were similarly followed by stabilization. The parallel trajectories between pre-slaughter (DOA) and post-slaughter (%condemnation) indicators reinforce the robustness of the adaptive response and highlight the integrator’s capacity to maintain animal health and product quality under ABF conditions.

### 4.3. The Implementation of Adaptive Management in ABF Systems

The estimated relative effect was associated with substantial uncertainty, as indicated by the wide credible interval (−38% to 657%) and the non-significant posterior probability. Although the posterior mean suggested a positive relative effect, the data did not provide strong evidence regarding either the magnitude or direction of the effect. Consequently, the results should be interpreted cautiously and should not be considered definitive evidence of an impact of the ABF transition on condemnation outcomes. The decline in %condemnation observed by 2020 suggests progressive adaptation of the production system to antibiotic-free (ABF) conditions. Rather than reflecting short-term fluctuations or seasonal effects, this improvement likely resulted from cumulative system-level adjustments that reduced disease susceptibility and improved carcass quality over time. Enhanced biosecurity and housing management may have limited pathogen exposure and transmission, while non-antibiotic nutritional interventions likely supported gut health and immune function, thereby reducing the incidence of pathological lesions leading to condemnation. Together, these effects are consistent with a gradual restoration of production stability following the initial ABF transition period, highlighting the capacity of integrated management strategies to mitigate ABF-associated risks without reliance on antibiotics [41].

Key interventions implemented on broiler farms in this study focused on strengthening biosecurity and optimizing environmental and nutritional management. Biosecurity measures were intensified through stricter farm entry control, enhanced hygiene practices, and improved sanitation of equipment and facilities. Non-antibiotic health supports, such as phytogenic additives, organic acids, and probiotics, were also adopted as part of farm management practices. In terms of biological explanation, improved environmental management helped minimize stress and respiratory issues in the flocks [36]. Strengthening water sanitation programmes helped maintain flock health by reducing biofilm buildup and limiting pathogen growth, often through the use of organic acids. Likewise, nutritional improvements that included probiotics, organic acids, and phytogenic compounds supported gut health and immunity, enhancing natural resistance to disease after antibiotic withdrawal [41,42,43].

Welfare-oriented measures reinforced these health-focused interventions. Stocking densities were maintained at ≤32 kg/m^2^ to minimize heat stress and competition for resources, and lighting programmes were adjusted to balance activity and rest [41], enhancing both comfort and immune function. Pre-slaughter stress was reduced through gentle handling, optimized transport and shorter lairage times, lowering the risk of injuries, bruising and stress-related carcase downgrades. Litter quality was managed through routine monitoring, timely replacement, and careful moisture control to sustain leg health and reduce skin lesions in a high-humidity tropical environment. The effectiveness of these welfare and management adaptations aligns with previous findings indicating that ABF production does not adversely affect flock health or processing losses compared with conventional systems, while showing improvements in housing conditions, health, and behavioural welfare indicators such as reduced hock burn, foot-pad dermatitis, lameness, and slightly lower mortality [3].

### 4.4. Implications for Industry and Policy

Although antibiotics historically played a central role in flock health [6], the results indicate that comparable outcomes are achievable under ABF systems when robust management, biosecurity, nutrition and welfare practices are in place. The stabilization of %condemnation after an initial adjustment period illustrates producer resilience and the adaptability of poultry systems in tropical conditions. Beyond welfare and health aspects, maintaining low condemnation rates under ABF production also carries important economic and sustainability benefits, as carcass rejection represents a direct financial loss. Reducing condemnation through improved management may improve resource efficiency and contribute to more sustainable poultry supply chains; however, the economic trade-offs associated with enhanced management under antibiotic-free production were not evaluated in this study. These findings provide practical evidence to guide producers, veterinarians and policymakers in supporting the transition from AB to ABF production without compromising processing performance, thereby advancing antimicrobial stewardship, economic efficiency, and responsible food production within sustainable poultry systems.

Although declining condemnation rates suggest improved health outcomes during the transition to antibiotic-free (ABF) production, implementation of ABF systems requires substantial investment in biosecurity, management, vaccination, and non-antibiotic alternatives, the feasibility of which may vary across farms. This study relied exclusively on slaughterhouse condemnation data; therefore, economic impacts, farm-level variability, and on-farm performance indicators (e.g., mortality, growth, disease pressure) were not assessed, representing a key limitation. Condemnation outcomes are influenced by multiple factors including flock characteristics, management practices, and processing conditions but consistent longitudinal data for these variables were unavailable. Consequently, the analysis was based on univariate slaughterhouse-level time-series data. Future studies should incorporate sensitivity analyses, multivariate models, and detailed farm-level and economic data to better isolate the effects of transitioning from antibiotic-based to ABF production. This study did not include a formal economic cost–benefit analysis comparing antibiotic-based and antibiotic-free production systems and therefore cannot assess whether the costs of enhanced management are offset by savings from reduced condemnation.

### 4.5. Study Limitations and Future Directions

Several limitations should be acknowledged. This study employed the BSTS framework using a univariable at the industry level based on weekly condemnation data aggregated from contact farms. Accordingly, the analysis relied on aggregated weekly observations rather than disaggregated farm-level data. Because the analysis was conducted using aggregated weekly data, variation among individual farms and truckloads could not be explicitly evaluated. Future studies using hierarchical or multilevel modelling approaches may provide additional insight into farm-level heterogeneity in condemnation outcomes. Management-related factors at the individual farm level, which likely vary across locations and over the six-year study period, were not incorporated due to the infeasibility of systematically collecting such data on a scale. Consequently, the analysis does not account for heterogeneity in farm management practices that may influence condemnation outcomes. As a result, the study may not fully support the identification or inference of specific farm-level risk factors, such as bird weight and age or other production-related variables that may be associated with condemnation outcomes, for which more detailed data collection at a finer scale and the application of alternative analytical approaches would be required. The analysis focused on total condemnation percentage; it was not possible to determine whether observed changes were primarily associated with infectious, metabolic, welfare-related, or processing-related causes of condemnation. Future studies examining individual condemnation categories may provide greater insight into the biological mechanisms associated with ABF production.

The present study employed a single BSTS specification implemented through the CausalImpact framework. Sensitivity analyses using alternative prior distributions or model structures were not performed; therefore, the robustness of the findings to alternative modelling assumptions remains to be investigated in future studies.

Moreover, because the findings were derived from a single production system operating under specific geographical and management conditions, the generalizability of the results to other contexts may be limited. Future research should therefore integrate flock-level health, welfare, and productivity indicators to provide a more comprehensive understanding of the broader impacts of ABF production and to identify management strategies that most effectively reduce condemnation risk while advancing sustainable poultry production.

## 5. Conclusions

BSTS analysis demonstrated that the ABF transition did not produce a statistically significant causal effect on %condemnation. The increase observed during the early ABF period likely reflected short-term adjustment effects inherent to the transition process. The transition to the ABF production system was not associated with a statistically significant change in condemnation percentage. However, given the observational nature of the study, the absence of a parallel control population, and potential unmeasured confounding factors, these findings should be interpreted cautiously and not as definitive evidence of causal effects. The subsequent decline demonstrates that stable processing outcomes can be maintained under ABF systems when supported by effective management, biosecurity, nutrition, and welfare practices. Implementation of the integrated ABF production programme was associated with changes in condemnation outcomes. Conducted under real-world commercial production conditions, this study provides empirical evidence from an operational-scale transition, illustrating that antimicrobial stewardship can be advanced without compromising processing performance. The findings of this study should be interpreted as associations observed following the transition to an ABF production system. Further research incorporating detailed management, health, and environmental data is needed to better understand the mechanisms underlying these observed patterns.

## Figures and Tables

**Table 1:** Summary of actual, predicted, absolute, and relative effects estimated by Bayesian structural time-series analysis for the 2018 transition to an antibiotic-free (ABF) rearing programme.

Variables	Average	Cumulative
Actual	3.90	609.10
Prediction (SD)	2.80 (1.30)	442.60 (208.40)
95% CI	(0.12, 5.50)	(19.35, 850.30)
Absolute effect (SD)	1.10 (1.30)	166.50 (208.40)
95% CI	(−1.50, 3.80)	(−241.20, 589.70)
Relative effect (SD)	95% (1090%)	95% (1090%)
95% CI	(−38%, 657%)	(−38%, 657%)

Posterior tail-area probability *p* = 0.207. Posterior probability of a causal effect = 79%. SD denotes standard deviation and CI is credible interval.

## Data Availability

The datasets generated and/or analyzed during the current study are not publicly available due to the data-sharing agreement with the commercial integrator but are available from the corresponding author on reasonable request and with permission from the data provider.
